# Study of Mild Steel Sandwich Structure Energy Absorption Performance Subjected to Localized Impulsive Loading

**DOI:** 10.3390/ma13030670

**Published:** 2020-02-03

**Authors:** Nouman Alqwasmi, Faris Tarlochan, Sami E. Alkhatib

**Affiliations:** 1 Department of Mechanical and Industrial Engineering, College of Engineering, Qatar University, P.O. 271, Doha, Qatar; na1205689@student.qu.edu.qa; 2 Qatar Transportation and Traffic Safety Center, College of Engineering, Qatar University, P.O. 271, Doha, Qatar; 3 School of Mechanical and Chemical Engineering, The University of Western Australia, 35 Stirling Highway, Crawley, Perth, WA 6009, Australia; sami.alkhatib@research.uwa.edu.au

**Keywords:** mild steel, compression blast loading, finite element analysis, thin-walled tube, sandwich structure

## Abstract

Extensive research focus had been given to sacrificial sandwich panels to mitigate the effects of blast loads. This is due to their ability to distribute the load and absorb a significant portion of the blast energy. This paper studies the behavior of sacrificial sandwich mild steel panels of axially oriented octagonal tapered tubular cores subjected to near-field impulsive blast. The deformation behavior and several assessment parameters consisting of the peak force, stroke efficiency, energy absorption and core efficiency were investigated using validated finite element analysis. The developed deformation modes were mainly influenced by the top plate and tube thickness. Tubes of a 5° taper performed unfavorably, exhibiting increased peak force and lower energy absorption. Panels of top plate thickness of 4 mm exhibited higher stroke efficiency as compared to panels of lower thickness. The top plate and tube thickness significantly affected energy absorption. An increase of 73.5% in core efficiency was observed in thick-plate panels as compared to thin-plate ones.

## 1. Introduction

Impulsive loadings can be the result of accidental chemical reactions in chemical plants or an intended explosive detonation by terrorist attacks. These events cause significant damage to infrastructure and more importantly threaten people’s safety. Impulsive loads are the result of an explosive shock wave and they involve high strain rates (10^3^–10^4^). When a structure undergoes an impulsive loading due to an explosion, strain hardening, inertia and strain-rate effects characterize the deformation [1]. Therefore, design for impulsive load energy absorption is very critical and needs specific consideration to assure the safety of people and infrastructure.

To that end, a lot of research has been conducted towards the testing of structures’ responses under impulsive loading conditions. Karagiozova et al. [1] studied the behavior of thin-walled tubes placed in an axial position where they underwent a transmitted explosive load through an attached mass. It was found that the initial compressive phase plays an important role in the deformation and energy absorption process. Paepegem [2] conducted experimental and numerical tests on the behavior of singular composite tubes and sacrificial cladding with an array of the same tubes as a core. He suggested using a rear and top skin with small masses but of high bending stiffness to minimize localized effects. As most research on axially blasted members was done on a small scale, Denny and Clubley [3] conducted full-scale experiments on I-beam steel columns to mimic real explosion events, such as in industrial accidents.

Among the many different solutions proposed in the literature, sacrificial panels had proven superior as blast load absorbers. A sacrificial panel, as defined by Yuen et al. [4], consists of three essential components: a load distributor top-plate, an energy absorbing-core and a rigid back-plate. Sacrificial panels were found to have a better blast-resistance capabilities over monolithic plates under small-scaled loadings [5]. Moreover, Theobald and Nurick [6] designed a novel lightweight sandwich panel with indented square hollow tubes. They studied various tube arrangements, aspect ratios, tubes and top-plate thicknesses, aiming for the highest buckling stability. The top-plate thickness had a significant effect on energy absorption, however a small effect on buckling stability. Theobald and Nurick [7] ran a more recent experimental and numerical study considering localized loading applied to the top plate. Despite the irregular buckling introduced at the core, the authors claimed a good agreement found between the experimental and the numerical data. Arora et al. [8] researched the behavior of composite sandwiched panels, with a foam core, subjected to impulsive load. The cores’ failure was found to change from compressive to shear once the impulse exceeds a threshold. Furthermore, an innovative panel with a honeycomb core design was developed numerically and examined by Jin et al. [9]. The author achieved an enhancement in the blast mitigation characteristics. Recent studies investigated panels of a dome-shaped tubular core of varying clearance [10,11]. On the other hand, foam fillings and transverse tubular cores in sandwiched assemblies were also investigated [4,12].

Since the top-plate; analogous to monolithic plates, is an essential component of a sacrificial panel, a full understanding of this component behavior is critical before proposing a new panel’s design. Chung et al. [13] reviewed the response of metal monolithic plates against blast loading. Their results indicated that different failure modes are determined by the extent of distributing the blast load across the receiving plate. Li et al. [14] studied the effect of flat and single-curved basalt/epoxy laminates against blast loading. They reported that plate thickness has a significant role in enhancing the blast load resistance.

A lot of research has been conducted on thin-walled tubes for a better understanding of the core elements in terms of energy absorption [15,16]. Witteman [17] conducted a numerical study of the influence of polygonal cross-sections on the behavior of thin-walled tubes subjected to dynamic crushing in axial and oblique loadings. Octagonal tubes had the highest energy absorption characteristics under axial and oblique loading. Tarlochan et al. [18] conducted a similar study, where tubes of hexagonal and octagonal cross-sections had the highest energy absorption characteristics under axial and oblique loading, respectively. Mamalis et al. [19] compared the buckling characteristics of octagonal tubes with square and circular tubes under axial collapse. They found that octagonal tubes displayed better energy absorption characteristics than square tubes and similar to that of circular ones. Rossi et al. [20] claimed that an increased number of walls in polygonal tubes, under axial crushing, resulted in higher energy absorption and smaller permanent displacement.

Core elements could be positioned in an axial or a lateral configuration. Axially loaded members were found to absorb ten folds more energy compared to laterally loaded structures [21]. However, buckling stability is a big concern in this configuration, particularly when the tubes are subjected to off-axis impacts. A viable solution is geometric modifications to tubular structures to control the buckling behavior and the peak force of the structure. Yuen and Nurick [22] investigated different imperfections to tubular structures and found that mid-span indentations and circular cutouts were the most conventional. Reid and Reddy [23] studied the behavior of single and double-tapered metal tubes under axial and oblique quasi-static and dynamic loadings. They found that tapered tubes could withstand oblique impact as effective as axial ones, in addition to having better stable buckling in off-axis impacts. Sami et al. [24,25] conducted a similar study on corrugated-tapered profiles under axial and oblique dynamic loads and found that tapering enhanced stability. Similarly, Wu et al. [26] investigated straight tubes of corrugated walls and found that it helps to reduce the peak force.

Due to the increasing expenses on running impact and impulsive experiments, along with the associated restrictions and risks, researchers rely on numerical methods such as finite element analysis (*FEA*). *FEA* is capable of providing information on the deformation behavior and the response of the system under investigation. Palanivelu et al. [27] studied the performance of single metal cans against blast loading, experimentally and numerically. Their results suggested using an array of metal cans, stacked axially in the core of a sacrificial panel for better performance. Ding et al. [28] used *FEA* to validate his analytical model of two degrees of freedom, predicting the response of a facade panel subjected to blast loading. *FEA* was also used to optimize systems that had been analyzed experimentally as conducted by Yao et al. [29] on internal blast chambers and Zhang et al. [30] on concrete-filled double-skin steel-tubes. Masi et al. [31] proposed a mere numerical approach to designing the protective devices of an airplane body, claiming that the obtained results are reliable for use.

The surveyed literature proved that octagonal tubes offer better energy absorption characteristics than tubes of other cross-sections [17,18,19,20]. Moreover, tubes of tapered profiles have proven advantageous under oblique loadings as compared to straight tubular structures [23,24,25]. Hence, a combined design of both characteristics (octagonal cross section and a tapered profile) might prove beneficial for impact applications.

To the authors’ knowledge, no research work had been carried out to design and test octagonal tapered tubes in a panel system for near-field blast loads. Therefore, this research aims to study the octagonal tapered profile along the length of thin-walled tubes, to enhance the energy absorption against near-field blasts. The buckling behavior and energy absorption performance of sacrificial sandwich panels with axially oriented octagonal tapered core tubes against near-field impulsive loading were examined numerically. Different configurations of panels were proposed and the finite element code Abaqus/Explicit was used to model and predict the behavior. The influence of the tube taper angle (*θ*), tube aspect ratio (*R*), tube thickness (*t*), top-plate thickness (*T*), cross-sectional ratio (*CR*) were investigated to understand the behavior of the tested panels under blast load.

## 2. Performance Indicators

**Figure 1:**
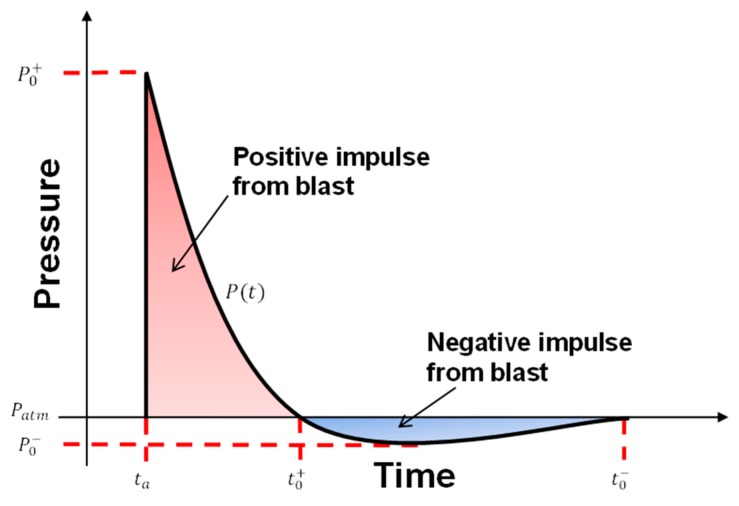
Ideal pressure-time profile of a blast event at a specific stand-off.

Whenever an explosive is detonated, a sudden shock front leads the propagating gas because of the pressure difference between the pressurized gas and the medium [32]. Once the shock front reaches the structure at point ‘*S*’ at a time of arrival ‘*t_a_*,’ a pressure impulse ‘*P_o_*^+^’ is created. Following *P_o_^+^* are two, positive and negative, phases of the explosion (Figure 1). The positive phase is ahead of the negative one with a duration of ‘*t_o_*^+^,’ rendering this phase an essential component in blast applications. The negative post phase goes below atmospheric pressure, reaching a minimum of ‘*P_o_*^‒^’ with a duration of ‘*t_o_*^−^.’ Although the negative phase has a longer period, it is insignificant in comparison to the positive phase.

The reflected impulsive pressure from a blast, measured at *S*, starts at *P_o_^+^* then reduces to *P_o_*^‒^ in an exponential form. Sacrificial panels work on reducing the force levels exhibited by the panel structure (i.e., protected) and extends the time of the impulse event. The forces following form local peaks and troughs, which interpret the formation of folds’ hinges on the crushed structure. Along, the following factors are used to assess the performance of the sacrificial panel, core tubes.

**Figure 2:**
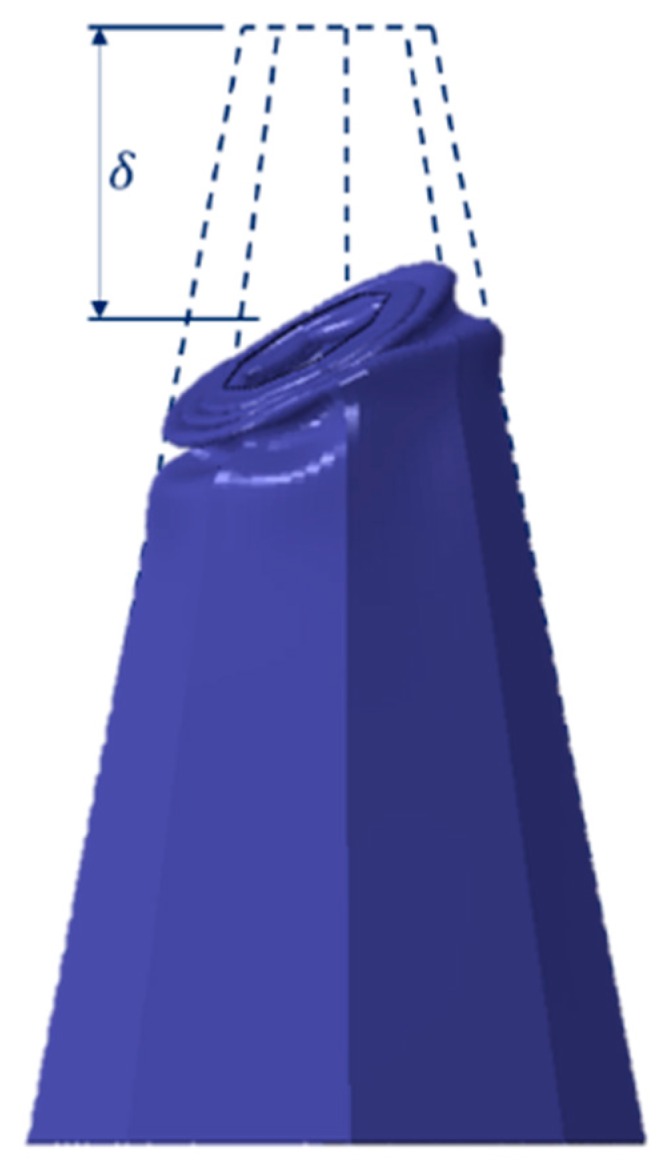
Mild steel deformed tube representation.

Generally, axially loaded thin-walled core tubes, in a sacrificial structure, undergo progressive or Euler buckling, among other deformation modes [33]. The deformation mode denotes the buckling stability, which highly influences the absorbed energy. The peak force corresponds to the elastic stress limit of core tubes, after which plastic buckling initiates. It also interprets the maximum transferred load to the non-sacrificial structure, neglecting any contact between the top plate and the back plate during crushing. The maximum mean crush distance, *δ*, is the difference between the initial and the final positions of the tube after deformation, as demonstrated in Figure 2. Stroke efficiency, *ε_stroke_*, is the ratio between δ and the initial length of tube, *L* (Equation (1)):

(1) $$\varepsilon_{Stroke}=\frac{\delta }{L}.$$

It gives an insight into the tube’s performance under crushing, showing the extent of its contribution to energy absorption. Energy absorbed by the core tubes is calculated by summing the areas under the load (*F*)—displacement (*u*) diagrams for all tubes in a panel (Equation (2)). Mean load (*P_mean_*) denotes the average value of the load exhibited by the tubes under crushing (*n_t_*). It is the ratio of the energy absorbed by tube to the maximum mean crush distance of the tube (Equation (3)).

(2) $$EA=\overset{n_{t}}{\underset{i=1}{\sum }}\int_{0}^{\delta_{i}}F_{i}\left(u\right)\;du$$

(3) $$P_{mean}=\frac{\sum_{i}^{n_{t}}P_{mean.i}}{n_{t}}=\frac{\sum_{i}^{n_{t}}\frac{EA_{i}}{\delta_{i}}}{n_{t}}.$$

## 3. Finite Element Model

**Figure 3:**
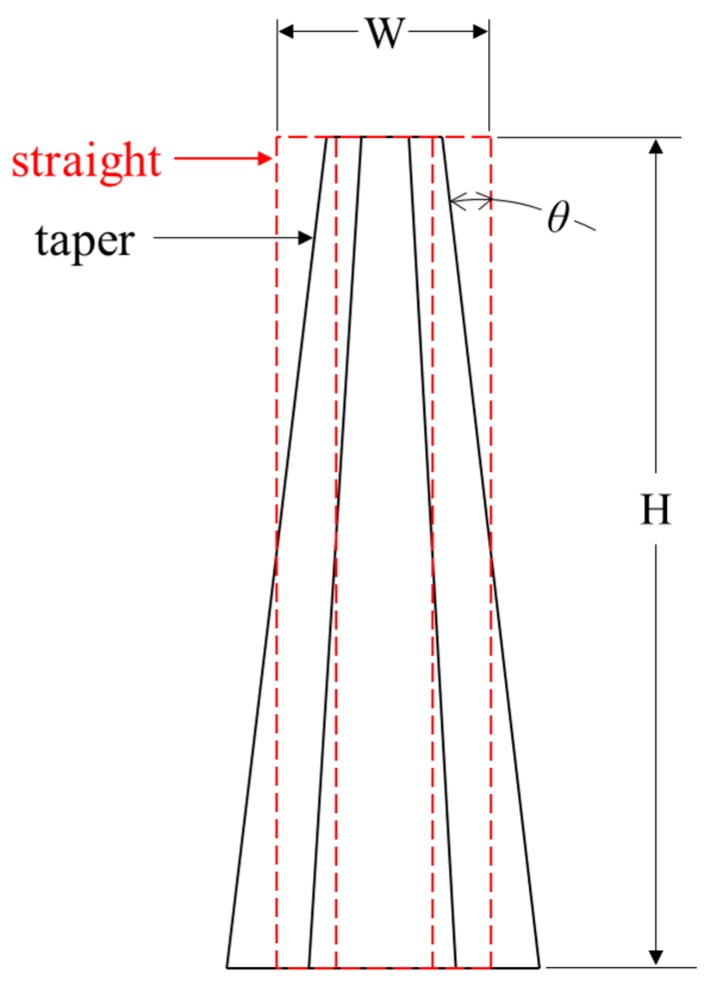
Mild steel straight to taper tube superimposition.

**Figure 4:**
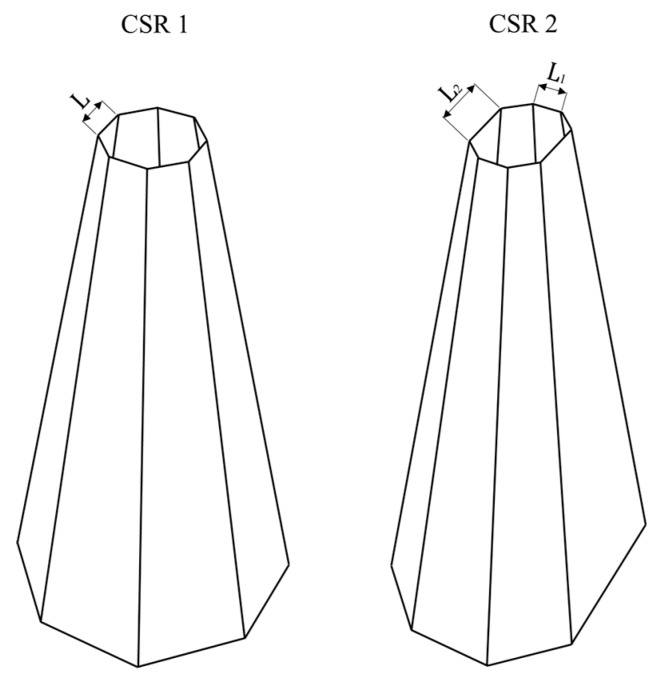
Tapered tubes design concept.

The proposed sandwich panel consists of a top plate, core tubes and a back plate. The design variables are the top plate thickness (*T*), cross-sectional ratio (*CR*), tube thickness (*t*), taper angle (*θ*) and the tube aspect ratio (*R*) and their values are listed in Table 1. Tubes’ mass was fixed by fixing *t* & *R*, hence, the mass allocation is a function of *θ* and *CR*. From a design point of view on mass allocation, the negative offset of the top face, while tapering, is equal in value to the positive offset of the bottom face (Figure 3 and Figure 4). For compact and feasible energy absorbing features, the tube’s length was set to 75 mm. Only four tubes were considered for the panel core, spread across a top plate of 150 mm × 150 mm × *T* mm.

**Figure 5:**
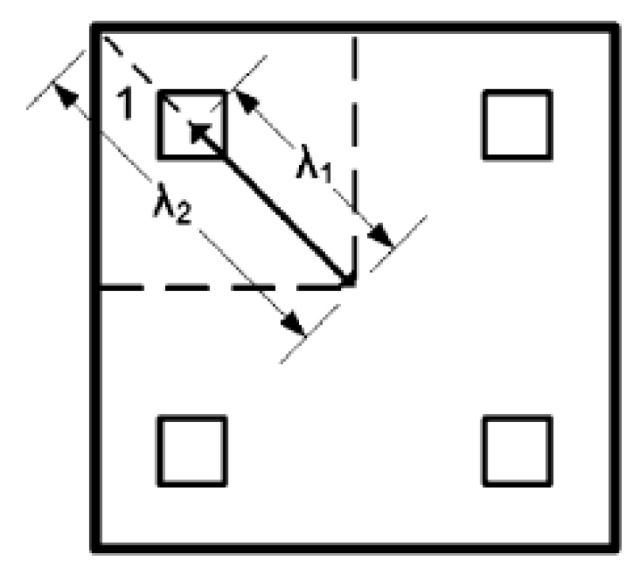
Top-view of panel design concept [2].

To assure buckling stability, the tubes were positioned in the core according to the ratio of the tube distance from the core center to half of the diagonal’s value, $\lambda =\frac{\lambda_{1}}{\lambda_{2}}$, as shown in Figure 5. The highest stability was achieved in the experiments with an optimum value of λ equal to 0.528 for the buckling of four core-tubes panels [6]. The core tubes were tied to both plates by the ‘tie constraint,’ with a friction coefficient of 0.3. Finally, a number notation of the form “*T–CR–t–θ–R*” was given to each panel.

**Figure 6:**
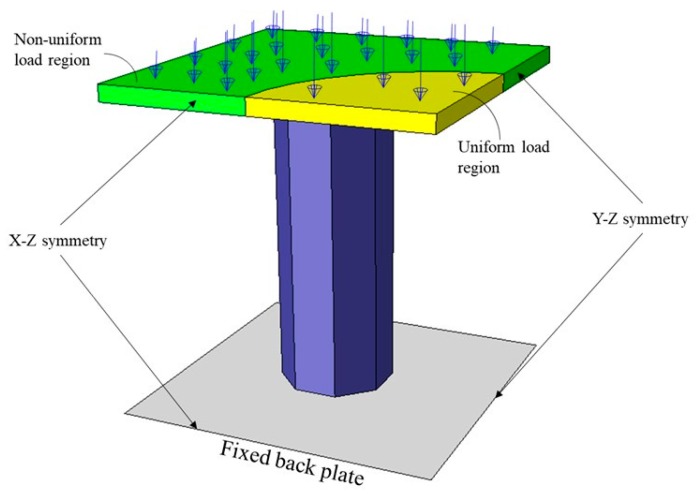
Quarter-symmetry of the numerical model boundary and load conditions.

Since the panel is symmetrical, only a quarter panel was simulated to reduce computation time (Figure 6). The tubes were meshed with ‘S4R’ 4-nodes reduced shell elements with five integration points. The top-plate was meshed with ‘C3D8R’ 8-nodes reduced cuboid elements with four elements across the thickness. The bottom-plate was meshed with ‘R3D4’ 4-nodes rigid element. To minimize the number of elements in the system, an optimum global size of 1 mm was assigned all the parts and 190 elements were assigned along the tubes’ length after conducting a mesh sensitivity study.

Reminnekov and Uy [34] differentiated between uniform and non-uniform blasts, clarifying that a scaled standoff distance (*Z*) exceeding a range of $1.2–2.0\frac{m}{{\mathrm{kg}}^{1/3}}$ corresponds to a uniform loading, while a small value within the range of $0.2–0.8\frac{m}{{\mathrm{kg}}^{1/3}}$ corresponds to a localized (i.e., non-uniform) loading. The value of Z can be calculated by:

(4) $$Z=\frac{R\prime }{\sqrt[{3}]{W}},$$

where *R*’ is the distance from the impulse and W is the explosive weight [32]. In a uniform loading, the pressure is distributed evenly across the structure’s face. In a localized loading, however, the pressure is centered which causes buckling instability. In this work we focused on localized loading to understand the effect of the proposed cores on stability.

The applied load on the panel was defined in terms of a localized pressure profile on the top plate by Theobald and Nurick [7]. The load profile is consistent for a specific centered radius and exponentially decays when moving radially outward beyond this radius. Since impulsive loading is adopted, the time-pressure distribution plays a less significant role than the spatial-pressure distribution. Therefore, the blast load is only assumed to be a function of spatial distance from the source. In polar coordinates convention, the pressure distribution on the top plate is as follows [7]:

(5) $$P\left(r,t\right)=\{\begin{array}{cc} P_{0}^{+} & r\leq a,\;0\leq t\leq t_{0}^{+}\\ P_{0}^{+}e^{-m\left(r-a\right)} & r>a,\;0\leq t\leq t_{0}^{+}\\ 0 & t>t_{0}^{+} \end{array},$$

where *r* is the radial distance from the top plate’s center, *a*, is the constant pressure radius and m is the decay constant. The value of *P_o_^+^* is a function of the positive impulse (I) [7]:

(6) $$P_{0}^{+}=\frac{I}{t_{0}^{+}\times \left[\pi \times a^{2}+8\int_{0}^{\pi /4\;}\left\{\frac{-e^{\frac{m\left(-w+acos\theta \right)}{cos\theta }}\times \left(mw-cos\theta \right)+\left(1+ma\right)\times cos\theta }{m^{2}\times cos\theta }\right\}d\theta \right]}$$

where *w* is the half width of the top plate with a prescribed value of 75 mm. Table 2 shows all the parameters needed to completely define the load profile. The blast duration value used is an estimate; however, a small difference in duration for a fixed impulse causes an insignificant effect on the panel’s response [7].

The material employed to all parts is mild steel with the true stress-strain values listed in Table 3. The rest of the mechanical properties are listed in Table 4 [7]. The strain-rate effect was accounted for using the Cowper-Symonds model that formulates the ratio of dynamic to static flow stress:

(7) σdσ0=[1+(ε˙plD)1q],

where $\sigma_{d}$ is the dynamic yield stress, $\sigma_{0}$ is the static yield stress, ${\dot{\varepsilon }}_{pl}$ is the plastic strain-rate and *D* and *q* are material constants. Finally, thermal softening was found to have an insignificant effect on the overall panel performance, therefore it was not accounted for to reduce the computation time [1].

**Figure 7:**
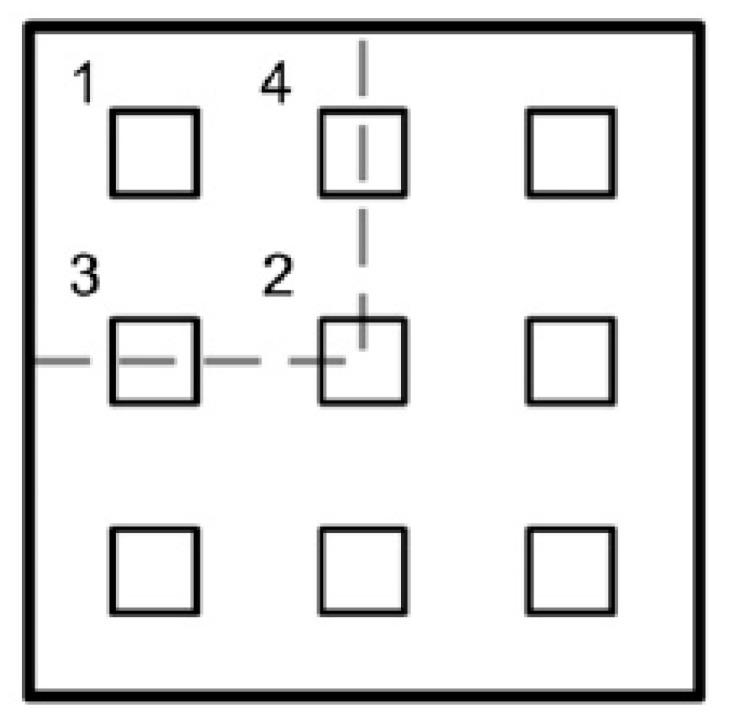
Nine core-tubes panel layouts [2].

The solver selected in ABAQUS was the build in the dynamic explicit. Here, an analysis time of 0.05 s was used because of the nature of the explosion. No mass scaling was used in the analysis. Automatic time adjustment was selected where ABAQUS/Explicit automatically adjusts the stable time increment during the analysis. Finite element (FE) model validation was carried out against published data [7] to assure the accuracy of the FEA results. A model of nine mild steel core-tubes of H of 75 mm, *t* of 0.61 mm and λ of 0.70 distributed evenly across the sacrificial panel (Figure 7), with a top-plate area of 150 mm×150 mm and a T of 4 mm. The blast pressure was modeled with a uniform profile with $P_{0}^{+}$ of 274 N/mm^2^:

(8) $$P\left(t\right)=\{\begin{array}{cc} P_{0}^{+} & 0\leq t\leq t_{0}\\ 0 & t>t_{0} \end{array}.$$

Equation (8) is a special case of Equation (5), where m is 0. The blast duration *t_o_* was set to 10 µs.

**Figure 8:**
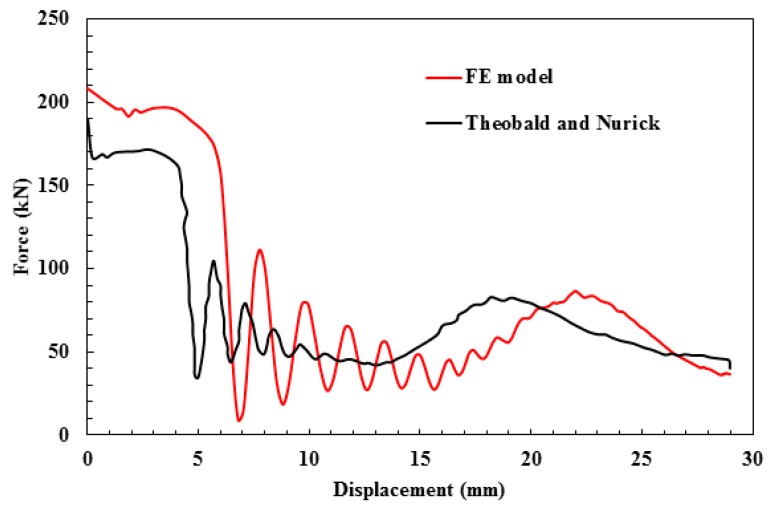
Force-displacement curve of current finite element (FE) model and Theobald and Nurick [7] for a panel of nine core tubes.

The force-displacement diagrams of the developed FE model and the published work [7] are mapped and plotted against each other in Figure 8. As observed, the course of progression of the force with displacement matches well with the published data. Furthermore, the assessment parameters of the peak force, crush distance and energy absorbed were extracted and compared in Table 5. The values were found within the acceptable margins to the published results. Therefore, the model was validated and can be used to predict the response of sacrificial panels of octagonal core tubes.

## 4. Results and Discussion

**Figure 9:**
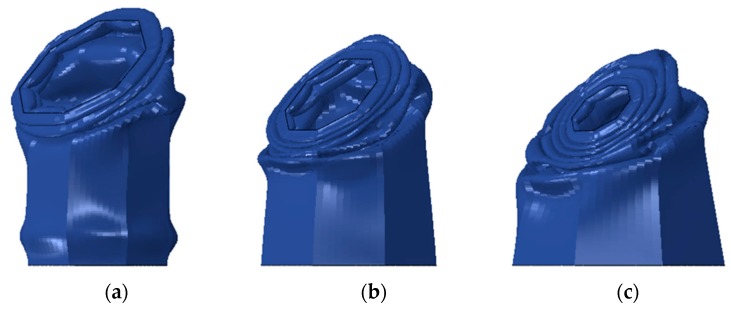
Crushed tubes of panels 4-1-0.6-θ-3 (**a**) *θ* = 0° (**b**) *θ* = 5° (**c**) *θ* = 10°.

**Figure 10:**
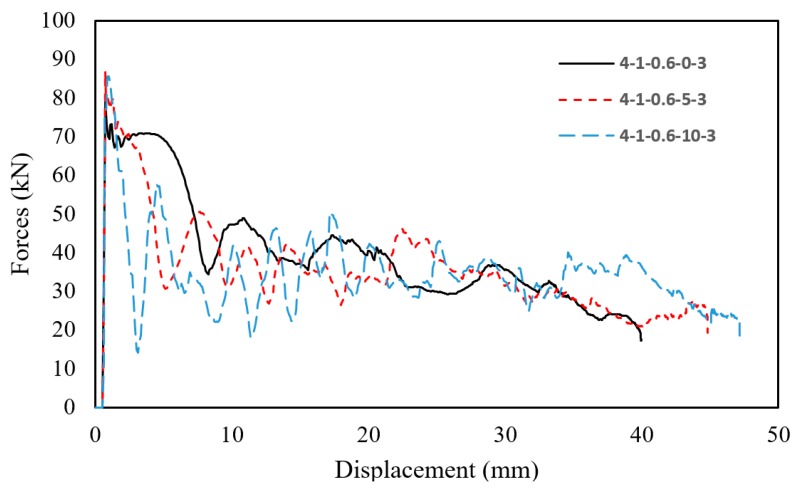
Plot of force-displacement curves of panels 4*–*1*–*0.6*–θ*–3.

**Figure 11:**
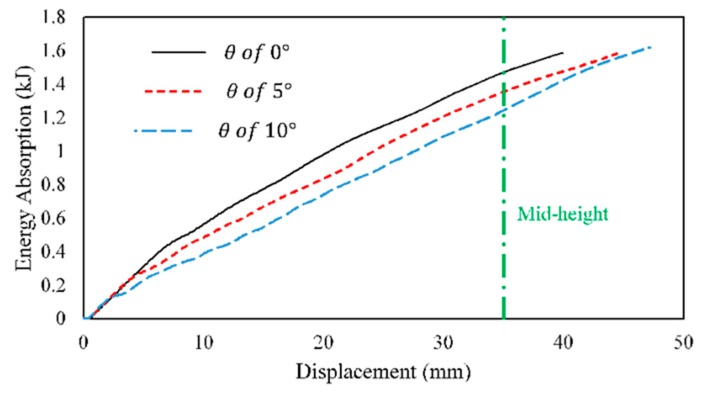
Plot of energy absorption-displacement curves of panels 4–1–0.6–*θ*–3.

Figure 9, Figure 10 and Figure 11 show typical crush behavior, force-displacement and energy absorption-displacement diagrams for sacrificial panels of *T* = 4 mm, *CR* = 1, *t* = 0.6 mm, *R* = 3 and varying *θ*. This was done to merely investigate the effect of θ on the performance of panels. From Figure 10, it is evident that increasing *θ* resulted in sustaining the force levels for a higher stroke, indicating a stable buckling. This is because of the tube’s tapered profile being more resistive to oblique loading that facilitates off-axis buckling. Moreover, an increase of crush distance was observed with an increasing *θ*. This is due to the low resistance in tapered tubes that corresponds to the smaller top diameter (i.e., inertia), which was a result of mass allocation while varying *θ*.

Additionally, the energy absorption-displacement curves showed a decrease in the slope of energy absorption with an increased *θ*, as shown in Figure 11. The energy absorption per buckled length (EA/L) was the highest for straight tubes (*θ = 0°*), up to the mid-length of the tubes (*δ* = 35 mm). After this point, the higher the *θ* led to a higher total EA and a crushed distance. The higher *EA/L* was attributed to the mass increase in the tube’s lower portion, corresponding to mass allocation. Likewise, the higher crush distance was attributed to initially buckling the small inertia of the upper portion of tube.

**Figure 12:**
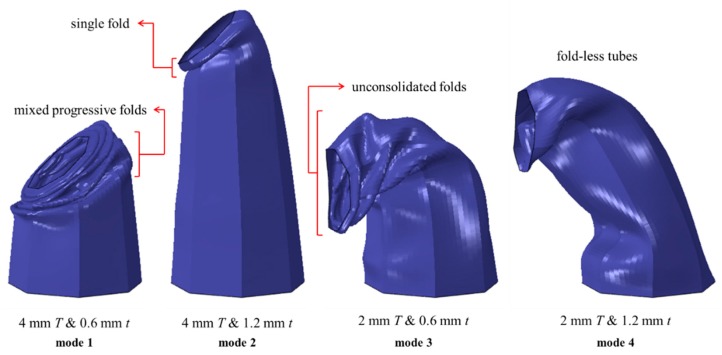
The four modes of deformation for tubes of *CR* = 1, *θ* = 5° and *R* = 3 and different *T* and *t*.

**Figure 13:**
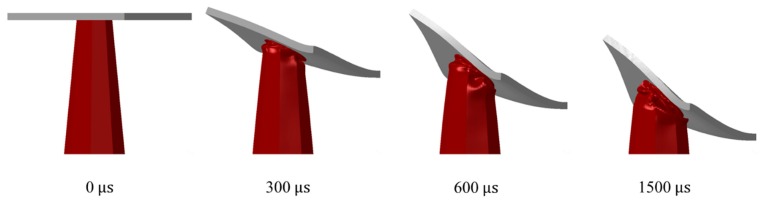
‘4–1–0.6–5–3’ panel deformation behavior with time.

The deformation mode implies tube’s behavior, fold’s shape, *EA* efficiency and stability during buckling. The core tubes exhibited four deformation modes, which are categorized utilizing different sets of plate and tube thickness as shown in Figure 12. Mode 1 was the most progressive deformation mode with mixed folds and a tilted tube top due to the oblique nature of loading. Mode 2 of deformation considered tubes with a single biased fold, pointing toward the load source. Since Modes 1 and 2 correspond to *T* of 4 mm, it was evident that plate contact was majorly avoided in panels under these modes (Figure 13).

**Figure 14:**
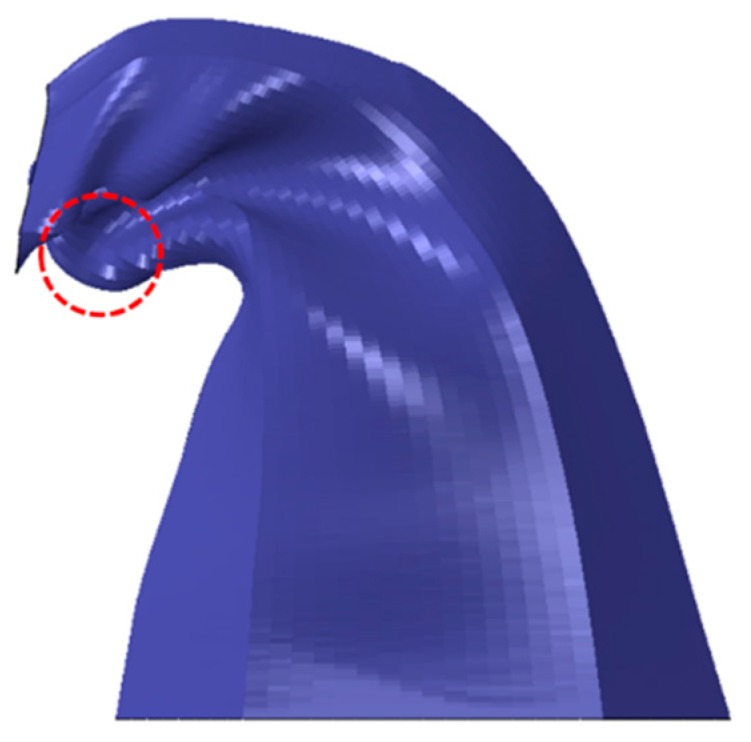
Fold-like bulge of core tube of panel ‘2–2–1.2–10–3’.

**Figure 15:**
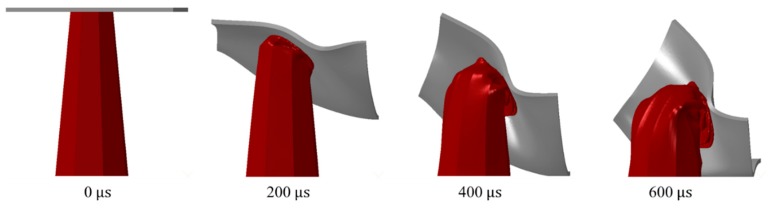
‘2–1–0.6–5–3’ panel deformation behavior with time.

Tubes with Mode 3 exhibited Euler buckling and formed a high number of folds, with most folds being unconsolidated and biased toward the panel’s center side. Unconsolidated folds result in insignificant energy absorption. Panels of Mode 4 were ‘the fold-less’ core tubes, with a middle flattening in the structure. Some special sets of *T* and *t* in Mode 4 exhibited fold-like bulges formed beneath the proximal end (Figure 14). All panels with a T of 2 mm (Mode 3 and 4) underwent Euler buckling, illustrated in Figure 15. Finally, tubes of *CR* of 2 were able to form one more hinge on average, as compared to tubes of *CR* of 1. This resulted in higher energy absorption for panels of *CR* of 2.

The geometrical parameters and the sacrificial panels’ responses are listed in the Appendix. A detailed analysis and explanation of the panels’ responses are presented in this section.

**Figure 16:**
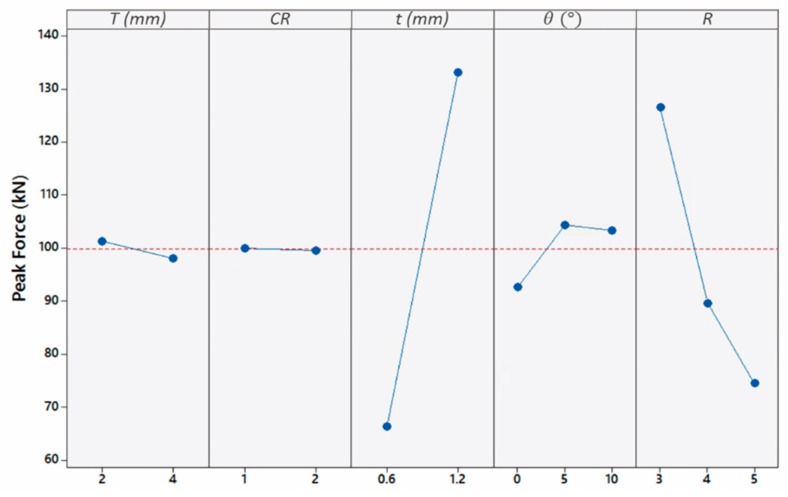
Influence of geometrical parameters on Peak Force.

Figure 16 shows the average effect of the geometrical parameters on the peak force. Such plot is generated by using the Minitab software based on the data obtained from the simulations. Based on the selected design of experiments, the response for each setup is entered into the design of experiment table in Minitab. Minitab helps by using in build functions to generate this plot. There is an insignificant decrease in peak force in thick top plates as compared to thin top plates. This decrease is because thin plates are associated with a smaller inertia; hence, they experience higher attained velocity. Therefore, strain-hardening effect is going to take part in the tube buckling. Furthermore, the aspect ratio is another parameter that influenced F significantly with a negative correlation. This is attributed to the less resisting material attributed to increasing the aspect ratio, against the load causing buckling. Similar behavior was reported in Reference [6] for straight tubes against blast loading. Conversely, *F* was found to almost double in value when doubling the tube thickness. This increase can be attributed to the stiffness increase resulting in a higher load to initialize crushing.

Likewise, the taper angle had an alternating influence on peak force. Unconditionally, the peak forces for all tubes with a taper angle of 5° were the highest, while they were lower for 0° and 10° with an absolute difference ranging from 1.46 kN to 15.12 kN. The reason behind this alternating behavior of taper angle shows a compromise between the effect of inertia, strain and strain-rate hardening. Tubes of a higher mass above mid-length are stiffer to initiate buckling (i.e., lower taper angle), because of higher inertia. Simultaneously, these tubes experience slower buckling, resulting in a smaller strain-rate hardening effect. As an example, panels with a top-plate of 4 mm, *CR* of 1, *t* of 0.6 mm and an *R* of 4, had a *F* of 57.24, 65.39 and 53.94 kN for a *θ* of 0°, 5° and 10°, respectively. Finally, it is shown that *CR* was the only geometrical parameter that had no effect on *F*.

**Figure 17:**
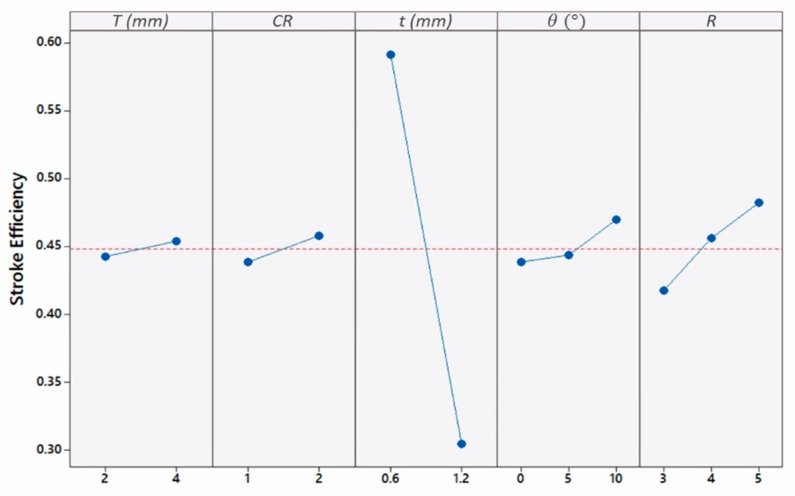
Influence of geometrical parameters on $\varepsilon_{stroke}$.

Since the tubes’ length was fixed, the average effect of geometrical parameters on stroke efficiency was plotted in Figure 17, instead of crush distance. It can be seen from Figure 17 that the tube’s *ε_stroke_* increased with the taper angle. In 5° taper tubes, the strain-rate hardening effect was higher than inertia effect, resulting in a higher resistance to deformation. Moreover, the aspect ratio was found to increase *ε_stroke_* in a near-uniform manner. It was deduced that the increase of *ε_stroke_* with aspect ratio was higher in panels with thicker top-plates than thinner ones, with an average of 0.06 and 0.02, respectively (Appendix). This is attributed to the general change in deformation mode when changing from a top-plate of 4 mm to 2 mm.

**Figure 18:**
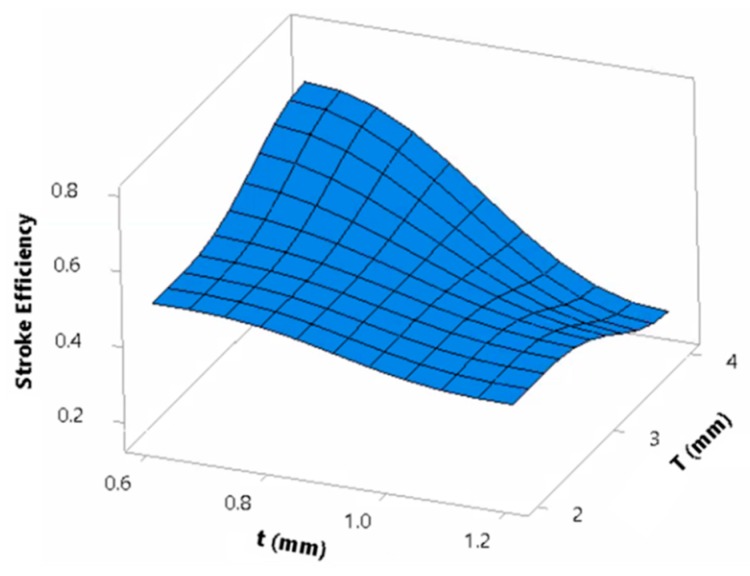
Dependence of $\varepsilon_{stroke}$ on the interaction of top-plate and tube thicknesses.

The top-plate thickness and tube thickness developed an interaction influence on $\varepsilon_{stroke}$ (Figure 18). The value of $\varepsilon_{stroke}$ for a top-plate thickness of 4 mm and tube thickness of 0.6 mm was the highest with an average of 0.68. The lowest value of $\varepsilon_{stroke}$ corresponded to a plate of 4 mm and a tube thickness of 1.2 mm, with an average of 0.23 mm. Panels with a plate thickness of 2 mm forced the tubes to deform excessively until plates contact occurred, resulting in a greater $\varepsilon_{stroke}$ value, although, the tubes underwent the Euler buckling mode. Because of the sufficient load distribution in thick plates, thin tubes contributed highly to the crushing. This finding conforms with the previous study [6] suggesting the use of thick plates for an idealized panel performance. Lastly, *CR* was found to cause a slight increase in $\varepsilon_{stroke}$. This could be a result of the narrower width in tubes with a *CR* of 2 as compared to 1 that caused the tubes to be unstable.

**Figure 19:**
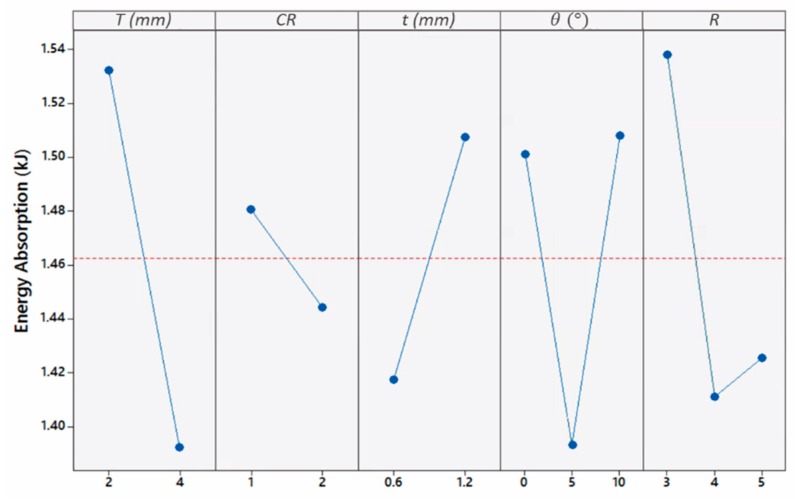
Influence of geometrical parameters on Energy Absorption.

The average effect of geometrical parameters on *EA* is depicted in Figure 19. From here, the energy absorption of the panels was highly influenced by the plate thickness, due to the deformation mode underwent by the different tube configurations. Because of its significance on *EA*, it is worth investigating *EA* individually for thin and thick plates panels. Once plates contacted in panels of thin tubes, *EA* decreased with the increase of taper angle and aspect ratio due to the corresponding stiffness decrease (Appendix). Conversely, thick tubes increased *EA* with taper angle and aspect ratio, although Euler mode of buckling was dominating. Hence, panels with a plate thickness of 2 mm and a tube thickness of 1.2 mm had the highest *EA* value with the leading panel being ‘2-2-1.2-0-5’ with a value of 2.34 kJ. This is because panels with a plate thickness of 2 mm forced thick tubes to crush completely similar to the thin tubes. The high stiffness of thick tubes allows for higher energy absorption as compared to their thinner counterparts.

**Figure 20:**
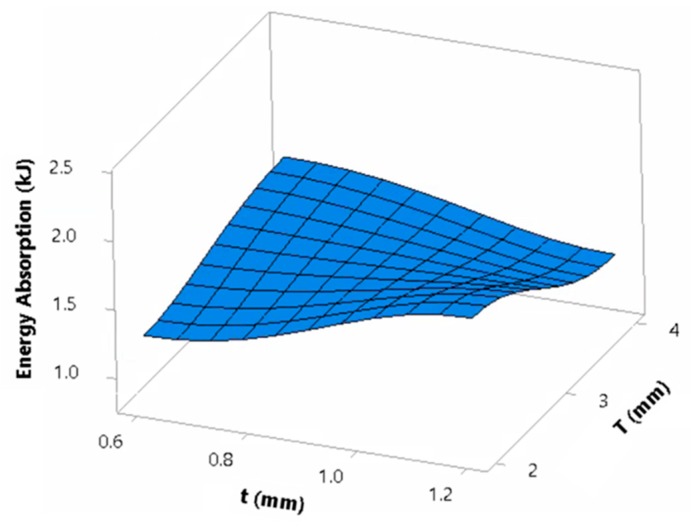
Dependence of Energy Absorption on the interaction of top-plate and tube thicknesses.

Moreover, tubes with a taper angle of 5° were found to absorb the lowest amount of energy for a plate thickness of 2 mm and a tube thickness of 1.2 mm. This result highlights the least favored effect a small taper holds on thin-walled energy absorbers under high strain-rate oblique loading. Furthermore, from a deformation point of view, tubes with a *CR* of 2 formed lower number of folds relative to other tubes of *CR* of 1, resulting in a decrease in *EA*. In contrast, there were some cases that found *CR* of 2 absorbing higher amounts of energy than tubes with a *CR* of 1. However, generally, no trend was found relating energy absorption to *CR*. The interaction plot in Figure 20 between top-plate and tube thickness summarizes the previously stated behavior of the panels in terms of *EA*. From here, it is observed in general that panels of a thin plate and a thick tube have the highest *EA* with an average of 1.79 kJ, while panels of a thin plate and a thin tube have the lowest *EA* with an average of 1.27 kJ.

**Figure 21:**
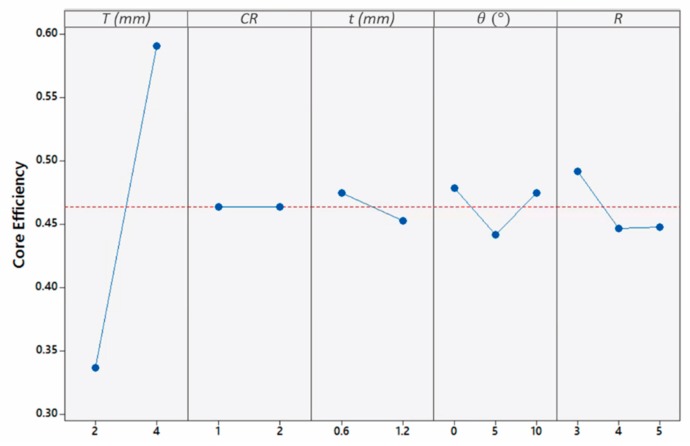
Influence of geometrical parameters on $\varepsilon_{core}$.

**Figure 22:**
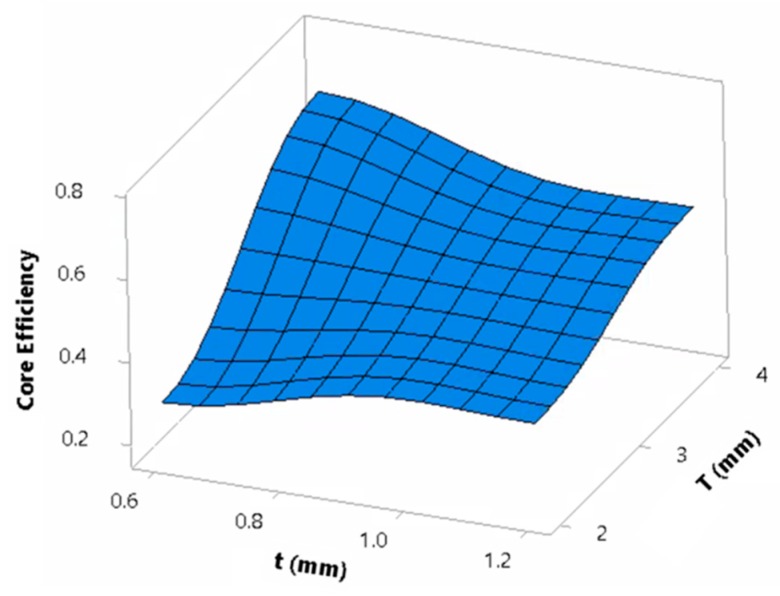
Dependence of $\varepsilon_{core}$ on the interaction of top-plate and tube thicknesses.

To further understand the panels’ behavior, the effect of geometrical parameters on core efficiency, ε_core_, is depicted in Figure 21. As shown in Figure 21, the influences of the top-plate and tube thickness on ε_core_ opposed the effect on *EA*, while the influences of taper angle and aspect ratio were similar. To elaborate the changed behavior of plate and tube thickness, the interaction of top plate and tube thickness is depicted in Figure 22. Unconditionally, thicker plates caused the panels to perform more efficiently than panels with thin plates in terms of ε_core_, with an increase of 73.5%. This is attributed to the higher level of buckling progression in thick plate’s panels as compared to that of the thin plate’s panels that suffer from Euler buckling. Similar findings were reported by researchers assessing thin-walled tubes subjected to compression [18,25]. Another attribute to the drastic fall of ε_core_ in thinner plate panels is the contact between the top and back plates. The contact will cause a very high amount of work to be applied to the non-sacrificial structure despite the higher energies absorbed by the core, leading to a decrease in ε_core_.

## 5. Conclusions

A numerical study using the finite element code Abaqus/Explicit was conducted to test the effect of the geometrical configurations on a sacrificial sandwich panel against near-field blasts. The panel cores were composed of axially oriented, octagonal cross-sectioned, straight and tapered tubes. The influences of tube taper angle (*θ*), tube aspect ratio (*R*), tube thickness (*t*), top-plate thickness (*T*) and the cross-sectional ratio (*CR*) were investigated. The force-displacement and energy absorption-displacement characteristics, deformation modes and a number of sacrificial panel assessment parameters were analyzed. Based on the analyzed geometrical parameters, the following conclusions were made:

- Tapered tubes were found to utilize higher stokes and a stable deformation behavior.
- Straight tubes achieved higher *EA* for their top half portion (before a mid-length of 35 mm); however, tapered tubes absorbed higher energy for their bottom portion, giving tapered tubes a slightly higher *EA* when employed in thick plates’ panels and lower *EA* in thin plates’ panels.
- The core tubes exhibited four deformations modes and they were influenced by the top-plate and tube thickness.
- With all assessment parameters taken into consideration, tubes with a 5 taper (i.e., small taper) performed unfavorably under high strain-rate oblique loading.
- Increasing the top-plate thickness and the aspect ratio and reducing the tube thickness result in higher.
- *CR* had minor to no influence on the assessment parameters, except on *EA* with no general trend.
- Panels with lower top-plate thickness and higher tube thickness (thick cores) resulted in higher *EA*, however stimulated top and back plate contact.
- An increase of 73.5% in core efficiency was observed for thick plates’ panels as compared to thin plate ones.

## Figures and Tables

**Table 1:** Panel geometrical parameters.

Design Variable	Equation	Values		
Top-plate thickness	*T*	2 mm		4 mm
Cross-sectional ratio	*CR* = $\frac{L_{1}}{L_{2}}$	1		2
Tube thickness	*t*	0.6 mm		1.2 mm
Taper angle	*θ*	0°	5°	10°
Aspect ratio	$R=\frac{H}{W}$	3	4	5

**Table 2:** Pressure distribution parameters.

*I* (Ns)	*a* (mm)	*m* (m^−1^)	$t_{o}\;(µs)$
50	40	40	10

**Table 3:** True stress-true plastic strain of mild steel [7].

$\sigma_{0}\;(\mathrm{MPa})$	223	230	246	260	275
ε_p_	0	0.042	0.088	0.148	0.25

**Table 4:** Mechanical properties of mild steel [7].

*ρ* (kg/m^3^)	*E* (GPa)	*υ*	*σ_yield_* (MPa)	*D* (s^−1^)	*q*
7850	200	0.3	287	2731	3.419

**Table 5:** Finite element model validation.

Reference	*F* (kN)	*δ* (mm)	*EA* (kJ)
Published data [7]	190	30	2.35
Current Study	208	29.44	2.32
Error (%)	9.5	1.86	1.29

## Appendix A

**Table A1:** Performance indicators data.

*T* (mm)	*CR*	*t* (mm)	*θ*	*R*	*L* (mm)	*F* (kN)	$P_{\mathrm{mean}}\;(\mathrm{kN})$	$\varepsilon_{stroke}$	*EA* (kJ)	$\varepsilon_{core}$
4	1	0.6	0	3	75	79.12	39.76	0.53	1.59	0.68
4	1	0.6	5	3	75	86.93	35.58	0.60	1.59	0.68
4	1	0.6	10	3	75	85.65	34.36	0.63	1.62	0.69
4	1	0.6	0	4	75	57.24	33.08	0.65	1.61	0.68
4	1	0.6	5	4	75	65.39	31.87	0.68	1.62	0.68
4	1	0.6	10	4	75	53.94	30.43	0.71	1.62	0.68
4	1	0.6	0	5	75	46.04	29.62	0.71	1.58	0.67
4	1	0.6	5	5	75	54.48	28.21	0.71	1.50	0.63
4	1	1.2	0	3	75	158.18	95.06	0.16	1.13	0.49
4	1	1.2	5	3	75	179.96	88.35	0.17	1.15	0.49
4	1	1.2	10	3	75	155.43	76.19	0.21	1.18	0.50
4	1	1.2	0	4	75	115.46	77.18	0.20	1.17	0.50
4	1	1.2	5	4	75	131.39	73.93	0.22	1.21	0.51
4	1	1.2	10	4	75	110.40	62.99	0.27	1.28	0.54
4	1	1.2	0	5	75	92.63	66.31	0.25	1.24	0.53
4	1	1.2	5	5	75	107.35	63.06	0.27	1.29	0.55
2	1	0.6	0	3	75	80.42	44.55	0.49	1.63	0.35
2	1	0.6	5	3	75	89.42	38.75	0.49	1.42	0.30
2	1	0.6	10	3	75	86.00	35.23	0.49	1.30	0.28
2	1	0.6	0	4	75	57.98	38.83	0.50	1.44	0.31
2	1	0.6	5	4	75	65.00	33.89	0.50	1.26	0.27
2	1	0.6	10	4	75	56.52	30.12	0.51	1.16	0.24
2	1	0.6	0	5	75	46.53	33.21	0.50	1.25	0.26
2	1	0.6	5	5	75	54.39	29.62	0.52	1.15	0.24
2	1	1.2	0	3	75	165.79	72.63	0.36	1.97	0.43
2	1	1.2	5	3	75	185.85	69.35	0.36	1.87	0.40
2	1	1.2	10	3	75	172.99	63.77	0.36	1.73	0.37
2	1	1.2	0	4	75	120.22	66.12	0.37	1.85	0.40
2	1	1.2	5	4	75	134.72	60.55	0.37	1.68	0.36
2	1	1.2	10	4	75	105.10	56.82	0.47	2.02	0.43
2	1	1.2	0	5	75	93.33	58.33	0.39	1.69	0.36
2	1	1.2	5	5	75	106.60	54.12	0.39	1.58	0.34
4	2	0.6	0	3	75	79.60	32.99	0.64	1.59	0.68
4	2	0.6	5	3	75	84.64	32.86	0.65	1.59	0.68
4	2	0.6	10	3	75	83.28	34.64	0.70	1.82	0.77
4	2	0.6	0	4	75	57.72	26.49	0.76	1.50	0.64
4	2	0.6	5	4	75	65.36	27.63	0.71	1.47	0.62
4	2	0.6	10	4	75	54.21	27.31	0.72	1.48	0.62
4	2	0.6	0	5	75	45.45	25.55	0.73	1.40	0.59
4	2	0.6	5	5	75	54.01	22.55	0.79	1.34	0.56
4	2	1.2	0	3	75	158.32	96.09	0.16	1.13	0.49
4	2	1.2	5	3	75	169.91	90.91	0.17	1.13	0.48
4	2	1.2	10	3	75	155.75	74.26	0.24	1.35	0.57
4	2	1.2	0	4	75	115.35	81.56	0.19	1.16	0.50
4	2	1.2	5	4	75	132.19	78.01	0.20	1.15	0.49
4	2	1.2	10	4	75	110.32	53.52	0.35	1.40	0.59
4	2	1.2	0	5	75	91.65	70.88	0.23	1.22	0.52
4	2	1.2	5	5	75	102.94	59.87	0.32	1.45	0.61
2	2	0.6	0	3	75	80.61	38.93	0.48	1.40	0.59
2	2	0.6	5	3	75	89.75	40.33	0.47	1.44	0.31
2	2	0.6	10	3	75	86.44	33.98	0.53	1.35	0.29
2	2	0.6	0	4	75	58.08	32.90	0.49	1.20	0.26
2	2	0.6	5	4	75	65.65	33.73	0.49	1.23	0.26
2	2	0.6	0	5	75	45.05	28.89	0.54	1.17	0.25
2	2	0.6	5	5	75	53.86	25.28	0.51	0.97	0.21
2	2	1.2	0	3	75	164.30	72.20	0.35	1.89	0.41
2	2	1.2	5	3	75	185.80	72.66	0.34	1.87	0.40
2	2	1.2	10	3	75	173.77	64.88	0.45	2.18	0.47
2	2	1.2	0	4	75	120.53	69.30	0.36	1.88	0.40
2	2	1.2	5	4	75	136.50	31.96	0.35	0.84	0.18
2	2	1.2	10	4	75	107.68	56.17	0.37	1.57	0.33
2	2	1.2	0	5	75	95.32	64.20	0.49	2.34	0.50
2	2	1.2	5	5	75	104.50	58.97	0.37	1.64	0.35
